# Factors associated with sarcopenia in elderly patients with type 2 diabetes mellitus

**DOI:** 10.3389/fendo.2026.1933664

**Published:** 2026-09-11

**Authors:** Ziqi Cao, Jiahui Li, Xinyu Gao, Wenzheng Tang, Anqi Zhang, Ziyu Ji, Yingshuai Li

**Affiliations:** Wangqi Academy, National Institute of TCM Constitution and Preventive Medicine, Beijing University of Chinese Medicine, Beijing, China

**Keywords:** associated factors, elderly patients with type 2 diabetes mellitus, meta-analysis, sarcopenia, systematic review

## Abstract

**Background and aims:**

Sarcopenia is a common geriatric syndrome that substantially impairs physical function, mobility, and quality of life in older patients with type 2 diabetes mellitus. However, evidence regarding factors associated with sarcopenia in this population remains fragmented and inconsistent, and previous studies have often included mixed-age populations or provided limited quantitative synthesis specifically focused on older adults. Therefore, this systematic review and meta-analysis aimed to comprehensively identify and quantify the associations between clinical factors and sarcopenia in older patients with type 2 diabetes mellitus, thereby providing age-specific evidence for clinical assessment, early identification, and individualized management.

**Methods:**

Relevant studies examining factors associated with sarcopenia in older patients with type 2 diabetes mellitus were systematically searched in six databases: PubMed, Web of Science, Embase, the Cochrane Library, CNKI, and Wanfang Data. The search covered the period from database inception to January 2026. Study characteristics, participant characteristics, potential associated factors, and sarcopenia-related outcomes were extracted. The methodological quality of the included cross-sectional studies was assessed using the AHRQ checklist. Meta-analyses were conducted using Review Manager version 5.3. Continuous variables were pooled using mean differences or standardized mean differences, and dichotomous variables were pooled using odds ratios. Publication bias and small-study effects were assessed using funnel plots and Egger’s regression test when at least 10 studies were available for a given outcome. For outcomes with substantial heterogeneity, REML-based 95% prediction intervals and exploratory univariable meta-regression analyses were conducted when sufficient data were available. The certainty of evidence for each pooled outcome was assessed using the GRADE approach.

**Results:**

A total of 19 cross-sectional studies involving 4,727 older patients with type 2 diabetes mellitus were included. The included studies were of moderate to high methodological quality, with no low-quality studies identified. Sarcopenia was diagnosed using different criteria, including the Asian Working Group for Sarcopenia 2014 criteria, the AWGS 2019 criteria, the European Working Group on Sarcopenia in Older People criteria, and the revised EWGSOP2 criteria. Meta-analysis showed that patients with sarcopenia were significantly older than those without sarcopenia, and had a longer duration of diabetes, higher fasting blood glucose levels, higher HbA1c levels, lower body mass index, and lower hemoglobin levels. No statistically significant associations were observed for male sex, total cholesterol, triglycerides, HDL-C, LDL-C, serum albumin, 25(OH)D, hypertension, smoking exposure, insulin therapy, or coronary/ischemic heart disease. Although sensitivity analyses generally supported the directional stability of the main findings, substantial heterogeneity remained for several outcomes, and all REML-based 95% prediction intervals crossed the null value. Exploratory meta-regression identified geographic region as a significant moderator only for HbA1c. Egger’s tests did not indicate statistically significant small-study effects for outcomes with sufficient studies. The certainty of evidence was rated as low for HDL-C, hypertension, and insulin therapy and as very low for all other pooled outcomes, primarily because of the cross-sectional study design, substantial inconsistency, and imprecision.

**Conclusion:**

In conclusion, older age, lower BMI, longer diabetes duration, higher fasting blood glucose, higher HbA1c, and lower hemoglobin levels were associated with sarcopenia in older patients with type 2 diabetes mellitus. However, the certainty of evidence was low to very low, and substantial heterogeneity and prediction intervals crossing the null value indicate that the magnitude and presence of these associations may vary across populations and clinical settings. These findings may heighten clinical suspicion during guideline-based sarcopenia assessment but should not be regarded as evidence of independent prediction, validated screening thresholds, or causality. Future prospective studies with standardized measurements and comprehensive adjustment for confounders are required.

**Systematic review registration:**

https://www.crd.york.ac.uk/prospero/, identifier CRD420261301123.

## Introduction

Type 2 diabetes mellitus (T2DM) is a common chronic metabolic disorder among older adults worldwide (1, 2). With the rapid aging of the global population, the prevention and management of diabetes-related complications have become an important public health challenge (3). Sarcopenia is an age-related, progressive disorder characterized by loss of skeletal muscle mass, reduced muscle strength, and impaired physical performance (4). It is associated with an increased likelihood of falls, fractures, disability, impaired quality of life, and adverse clinical outcomes in older adults (5).Previous studies have reported that sarcopenia is more prevalent among elderly patients with T2DM than among the general elderly population (6). The coexistence of T2DM and sarcopenia may be accompanied by interacting metabolic abnormalities, muscle deterioration, and functional decline, thereby increasing the complexity of clinical management.

Chronic hyperglycemia, insulin resistance, systemic inflammation, and other metabolic abnormalities may be involved in the association between T2DM and sarcopenia by disrupting the balance between muscle protein synthesis and degradation and by impairing muscle microcirculation, mitochondrial function, and neuromuscular integrity. Although numerous studies have investigated factors associated with sarcopenia in elderly patients with T2DM, including demographic characteristics, metabolic indicators, comorbidities, and hematological and nutritional parameters, the available evidence remains fragmented and inconsistent. Several systematic reviews and meta-analyses have evaluated factors associated with sarcopenia in patients with T2DM, but their findings have not been entirely consistent or comprehensive (7, 8). For example, age, diabetes duration, fasting blood glucose, and glycated hemoglobin have been reported to be associated with sarcopenia in some studies, whereas the direction and magnitude of the associations involving lipid components, hypertension, and other clinical indicators remain uncertain. Moreover, previous meta-analyses often included adults across broad age ranges rather than focusing specifically on elderly populations and did not comprehensively synthesize several clinically relevant continuous indicators, such as fasting blood glucose, low-density lipoprotein cholesterol, and hemoglobin (9).

Therefore, a systematic synthesis of the available evidence specifically concerning elderly patients with T2DM is warranted. In accordance with the Preferred Reporting Items for Systematic Reviews and Meta-Analyses (PRISMA) guidelines, the present systematic review and meta-analysis aimed to identify and quantify the associations between selected clinical factors and sarcopenia in elderly patients with T2DM. The findings are expected to provide age-specific evidence for clinical assessment, early identification, and individualized management. Given the observational and predominantly cross-sectional nature of the included studies, the identified associations should not be interpreted as evidence of causality.

## Methods

This systematic review and meta-analysis was conducted and reported in accordance with the Preferred Reporting Items for Systematic Reviews and Meta-Analyses (PRISMA) 2020 guidelines (10). The study protocol was prospectively registered with the International Prospective Register of Systematic Reviews (PROSPERO; registration number: CRD420261301123). Two reviewers independently performed the literature search, study screening, eligibility assessment, data extraction, and methodological quality assessment. Any disagreements were resolved through discussion or consultation with a third reviewer. Standardized procedures were applied for data synthesis and statistical analysis to enhance the reproducibility and reliability of the findings.

### Search strategy

Two independent researchers performed a comprehensive literature search in PubMed, Web of Science, Cochrane Library, Embase, CNKI, and Wanfang databases from database inception to January 2026 The search strategy was as follows: (“type 2 diabetes mellitus” OR “type 2 diabetes” OR “T2DM” OR “NIDDM”) AND (“elderly” OR “aged” OR “older adults” OR “older people” OR “geriatric”) AND (“sarcopenia” OR “sarcopenic” OR “muscle loss” OR “low muscle mass”) AND (“risk factors” OR “risk factor” OR “predictors” OR “determinants” OR “associated factors” OR “correlates”). Both text words and subject headings were used in the search. In addition, a manual search of the reference lists of the included studies and relevant reviews was conducted to supplement the literature search. The search was restricted to publications in Chinese or English because these were the languages that could be reliably assessed for eligibility and data extraction by the review team. Grey literature, including conference proceedings, dissertations, and other unpublished sources, was not systematically searched.

### Inclusion and exclusion criteria

This systematic review and meta-analysis was conducted in accordance with the PRISMA 2020 guidelines and the MOOSE (Meta-analysis of Observational Studies in Epidemiology) statement (11). The inclusion criteria were as follows: (1) Population: Patients with a confirmed diagnosis of type 2 diabetes mellitus who were aged ≥60 years. In this review, ≥60 years was prespecified as the operational threshold for older adulthood. Studies using a higher minimum age threshold, such as ≥65 years, were also eligible because all participants in these studies met the prespecified minimum age criterion. We acknowledge that chronological definitions of older adulthood are not uniform across countries, regions, and healthcare systems. Therefore, the minimum age criterion used in each study was extracted and its potential influence on the pooled findings was examined in sensitivity analyses. Studies involving broader age ranges were eligible only when data for participants aged ≥60 years could be extracted separately; (2) Exposure/Study Factors: Exploration of potential factors related to sarcopenia, including but not limited to demographic indicators, metabolic indicators, muscle function/morphology indicators, blood indicators, and comorbidities; (3) Outcome Indicators: Sarcopenia as the outcome, with clear diagnostic criteria; (4) Study Design: Observational studies (cohort studies, cross-sectional studies, case-control studies) that provide effect sizes and 95% confidence intervals (e.g., MD, SMD, RR(Relative Risk), OR(Odds Ratio), HR(Hazard Ratio), etc.); (5) Literature Type: Original studies published in Chinese or English, with complete full texts and extractable data.

The exclusion criteria were: (1) Excluded Study Types: Reviews, commentaries, conference abstracts, case reports, animal experiments, cell experiments, single-arm studies without a control group, and systematic reviews/meta-analyses; (2) Excluded Study Populations: Non-elderly patients (< 60 years old or studies without a clear definition of “elderly”), patients with type 1 diabetes or other types of diabetes, and patients with severe diseases affecting muscle metabolism (e.g., severe liver/kidney failure, malignant tumors, severe infections); (3) Excluded for Incomplete Data: Studies without clear sarcopenia diagnostic criteria, studies not reporting effect sizes and 95% CIs for the association between examined factors and sarcopenia, studies with irreplaceable missing data, and duplicate publications; (4) Studies with an AHRQ score ≤ 3 points (low quality).

### Literature screening and data extraction

A two-stage screening process was conducted by two independent reviewers (Cao Ziqi and Li Jiahui). In the initial deduplication stage, literature records were evaluated based on titles, publication years, and author metadata. After deduplication, the same reviewers performed a secondary eligibility assessment, systematically examining the titles, abstracts, and full texts of the remaining records. Disagreements at any stage were resolved through arbitration by a third party (Li Yingshuai) to reach a consensus.

The following information was extracted from each included study: first author, publication year, country or region, study design, data source, participant characteristics, total sample size, sample sizes of the sarcopenia and non-sarcopenia groups, diagnostic criteria for type 2 diabetes mellitus and sarcopenia, and methodological quality. In accordance with the review protocol, we sought to extract all reported factors potentially associated with sarcopenia rather than restricting data collection to the indicators ultimately included in the meta-analysis. These factors were categorized into demographic and anthropometric characteristics, lifestyle and behavioral factors, nutritional factors, diabetes-related clinical and treatment factors, metabolic and hematological indicators, comorbidities, and muscle-related measures. Particular attention was given to physical activity or exercise level, dietary protein intake, vitamin D status, insulin use or other glucose-lowering regimens, longitudinal glycemic measures where available, and skeletal muscle mass indices such as the skeletal muscle index or appendicular skeletal muscle mass index.

For continuous variables, the sample size, mean, and standard deviation of each group were extracted; for dichotomous variables, the number of events and the total number of participants in each group were recorded. The quantitative meta-analyses were primarily based on unadjusted group-level data. Adjusted effect estimates and their 95% confidence intervals were also collected when available; however, they were not quantitatively pooled because they were inconsistently reported across studies and were derived from substantially different covariate-adjustment models. Instead, adjusted estimates were summarized narratively when relevant. Quantitative synthesis was performed only when the factor was reported by a sufficient number of studies and when its definition, measurement method, and data format were considered clinically and methodologically comparable. Factors that could not be pooled because of insufficient data, incompatible reporting formats, or substantial differences in measurement methods were summarized in a **Supplementary Table** and synthesized narratively. All extracted data were independently checked by Li Jiahui to ensure accuracy and consistency.

### Quality assessment

The methodological quality of the included cross-sectional studies was evaluated using the AHRQ cross-sectional study assessment tool (12). This tool includes 11 items, with each item scored 1 point for “yes” and 0 points for “no” or “unclear” (total score: 0–11). Studies scoring 8–11 were considered high quality, 4–7 moderate quality, and 0–3 low quality. Quality assessment was performed independently by two assessors (Cao Ziqi and Li Jiahui) using this structured tool. Disagreements between assessors were resolved through consensus discussions facilitated by a third researcher (Li Yingshuai) to ensure standardized interpretation of the methodological rigor of the included studies. A total of 19 cross-sectional studies were included.

### Statistical analysis

Data were extracted using standardized forms and analyzed with Review Manager 5.3. Before quantitative synthesis, the definitions, measurement methods, and units of each variable were examined and harmonized where possible. Mean differences (MDs) were used for continuous variables measured using comparable methods and units, whereas standardized mean differences (SMDs) were applied when measurements could not be reasonably standardized. Odds ratios (ORs) were used for dichotomous variables. All effect estimates were reported with 95% confidence intervals (CIs). Quantitative meta-analysis was conducted only when at least two studies were sufficiently comparable in terms of study population, variable definition, and data reporting. When quantitative pooling was inappropriate because of an insufficient number of studies or substantial differences in definitions, units, reporting formats, or measurement methods, the available evidence was synthesized narratively. Factors that were reported but could not be quantitatively pooled were distinguished from factors that were not reported in the included studies. For narratively synthesized factors, individual study results, including effect estimates, 95% CIs, P values, and whether the estimates were adjusted or unadjusted, were extracted and summarized in a structured **Supplementary Table**. Findings were interpreted cautiously in view of differences in measurement methods, diagnostic criteria, and study populations.

Heterogeneity was assessed using Cochran’s Q test and the I^2^ statistic. The model-selection criteria were prespecified. A fixed-effect model was used when studies were sufficiently comparable and statistical heterogeneity was low (I^2^ ≤50% and Cochran’s Q-test P≥0.10); otherwise, a random-effects model was applied. Leave-one-out sensitivity analyses were performed, and exploratory subgroup analyses based on the AWGS 2014 and AWGS 2019 criteria were conducted when sufficient data were available. In addition, to evaluate whether differences in the operational definition of older adulthood influenced the pooled estimates, threshold-based sensitivity analyses were performed for each outcome that included studies restricting eligibility to participants aged ≥65 years. These studies were simultaneously excluded, and the resulting effect estimates, 95% CIs, statistical significance, and heterogeneity statistics were compared with those of the primary analyses. Outcomes containing no studies with a ≥65-year eligibility threshold were not reanalyzed. The same prespecified model-selection criteria were applied to these sensitivity analyses.

For outcomes including at least 10 studies, funnel plots and Egger’s test were performed using Stata 19.5 to assess potential publication bias and small-study effects. A P value <0.05 was considered suggestive of small-study effects. The trim-and-fill method was planned as an exploratory analysis when significant funnel plot asymmetry or small-study effects were detected. Publication bias was not assessed for outcomes with fewer than 10 studies or those synthesized narratively. For outcomes with substantial or clinically important between-study heterogeneity, restricted maximum likelihood random-effects models were fitted in Stata version 19.5 to calculate 95% prediction intervals.These approaches align with methodological recommendations for quantifying uncertainty in pooled estimates intended to inform prognostic inference across heterogeneous study settings (13–15). Exploratory univariable meta-regression analyses were performed when sufficient studies and adequately populated moderator categories were available. Sarcopenia diagnostic criteria and geographic region were treated as categorical moderators, whereas study-level mean age was treated as a continuous moderator. Each moderator was examined separately because of the limited number of included studies. REML estimation with Knapp–Hartung adjustment was used, with AWGS 2014 and China selected as the reference categories. Study-level mean age was not examined as a moderator for the age outcome because age itself was the outcome variable. Meta-regression was not performed for fasting blood glucose, hemoglobin, serum albumin, or 25(OH)D because the limited number of studies was considered insufficient to provide reliable moderator estimates. Given the exploratory nature of these analyses and their reliance on aggregate study-level data, we acknowledge that identified moderator associations cannot be directly extrapolated to individual-level predictions without further temporal validation and external testing (16, 17). Multivariable meta-regression was not performed because of the limited number of studies available for each outcome. The pooled estimates and forest plots generated in Review Manager were retained as the primary analyses, whereas the REML models in Stata were used specifically for exploratory meta-regression and calculation of 95% prediction intervals.

### Certainty of evidence assessment

The certainty of evidence was assessed separately for each pooled outcome using the GRADE approach. Because the aim of this review was to evaluate associations rather than causal effects, the target of the certainty assessment was the confidence in the estimated associations between the examined clinical factors and sarcopenia. This distinction reflects contemporary guidance on predictive risk assessment frameworks, which emphasizes that confidence in estimated associations from observational data should be evaluated separately from claims of causality or validated screening thresholds (18, 19). As all included studies were cross-sectional observational studies, the initial certainty was rated as low. Further downgrading was considered for serious concerns regarding risk of bias, inconsistency, indirectness, imprecision, and publication bias. Potential upgrading factors, including a large magnitude of association, a dose-response gradient, and the possible influence of residual confounding, were also considered where applicable. The reasons for each certainty judgment were documented in the Summary of Findings table. The final certainty of evidence was categorized as high, moderate, low, or very low.

## Results

### Literature selection and study characteristics

**Figure 1:**
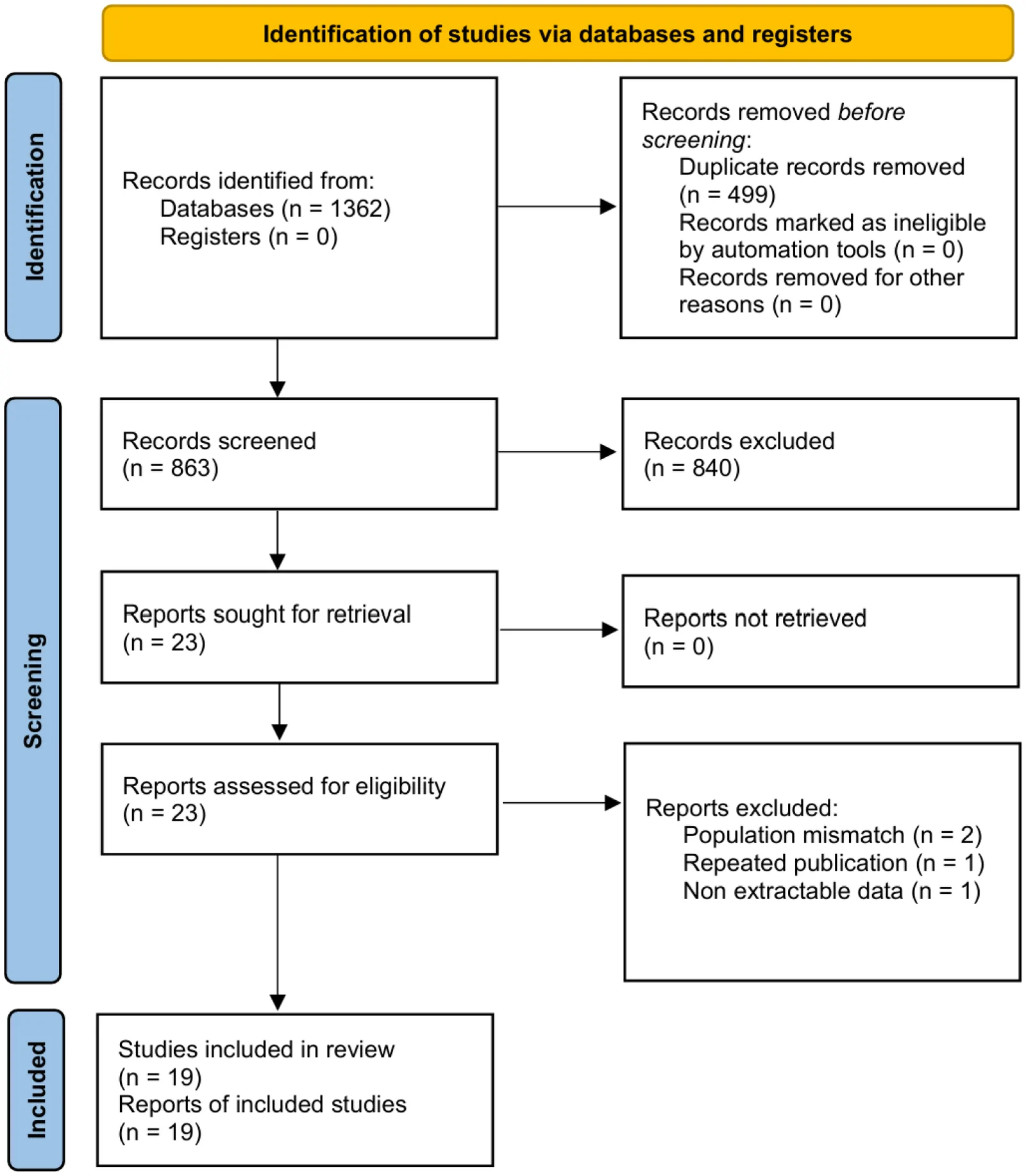
The PRISMA flow diagram displays the details of the selection process.

The PRISMA flow diagram (**Figure 1**) illustrates the study selection process. A total of 1,362 records were retrieved from six databases. After title and abstract screening, 23 articles were assessed for full-text eligibility. Following full-text evaluation, 19 studies were ultimately included in the systematic review. All included studies were used for qualitative synthesis, and outcome-specific meta-analyses were performed when sufficiently comparable data were available.

The 19 included studies were published between 2018 and 2025, all of which were cross-sectional studies involving 4,727 participants, including 1,262 patients with sarcopenia and 3,465 patients without sarcopenia. No eligible cohort or case-control studies were identified. The included studies were derived from CNKI, Wanfang, PubMed, and Embase. All participants were older adults with type 2 diabetes mellitus, Thirteen studies enrolled participants from 60 years of age onward, including studies with an upper age limit, whereas six studies restricted eligibility to participants aged ≥65 years. The study-specific age criteria are presented in **Table 1**. The diagnostic criteria for sarcopenia varied across studies: eight studies used the Asian Working Group for Sarcopenia 2014 criteria (AWGS 2014), eight used the AWGS 2019 criteria, two used the European Working Group on Sarcopenia in Older People criteria (EWGSOP1), and one used the revised EWGSOP2 criteria. Muscle mass was assessed using different methods across studies, predominantly bioelectrical impedance analysis (BIA), while several studies used dual-energy X-ray absorptiometry (DXA), and a small number also used anthropometric measures such as calf circumference. Although some included studies reported multivariable-adjusted association estimates, these estimates were inconsistently available and were based on different covariate-adjustment models. Therefore, they were not quantitatively pooled. The primary meta-analyses were based on unadjusted group-level data, whereas adjusted estimates were summarized narratively when relevant. The detailed diagnostic criteria, group sizes, and basic characteristics of the included studies are presented in **Table 1**.

**Table 1:** Characteristics of the included studies.

Author, year	Country/region	Database/source	Study design	Study setting	Sample size, n	Sarcopenia / nonsarcopenia, n	Age criterion	Sarcopenia diagnostic criteria	Muscle assessment method	Main reported associated factors	AHRQ score	Quality level
Yangna et al (20), 2025	China	CNKI	Cross-sectional	Hospital-based; Department of Geriatrics, Yuncheng Central Hospital	122	28 / 94	≥60 years	AWGS 2014	Body composition analyzer for ASM/ASMI; handgrip strength; walking speed/gait speed test	Demographic, anthropometric, glycemic, lipid, vitamin D, lifestyle	7	Moderate
Lichun et al (21), 2025	China	CNKI	Cross-sectional	Hospital-based; the 964 Hospital of the PLA Joint Service Support Force	380	168 / 212	≥60 years	AWGS 2019	BIA using InBody S10; ASMI	Demographic, anthropometric, glycemic, hematological/nutritional, lifestyle	8	High
Wanying et al (22), 2024	China	CNKI	Cross-sectional	Hospital-based; Qinhuangdao First Hospital	113	29 / 84	≥65 years	AWGS 2019	BIA using InBody 720; ASMI; handgrip strength; 6-m gait speed	Demographic, glycemic, vitamin D, lifestyle	7	Moderate
Zhangtian et al (23), 2019	China	CNKI	Cross-sectional	Hospital-based; inpatient patients from the Department of Endocrinology and Department of Geriatrics, Jiangyin People’s Hospital	280	85 / 195	≥60 years	AWGS 2014	BIA for body composition and ASMI; Jamar handgrip strength; 6-m gait speed	Demographic, anthropometric, diabetes duration, glycemic, vitamin D	7	Moderate
Yanan et al (24), 2019	China	CNKI	Cross-sectional	Outpatient-based; endocrinology clinics of two hospitals in Nantong	430	95 / 335	≥60 years	AWGS 2014	BIA for ASMI; handgrip strength; 6-m gait speed	Demographic, anthropometric, glycemic, medication-related	8	High
Yanling et al (25), 2024	China	Wanfang	Cross-sectional	Hospital-based; Department of Geriatrics, Wuhan Hanyang Hospital	241	55 / 186	≥60 years	AWGS 2019	BIA using InBody 770; ASMI; handgrip strength; SARC-F; gait speed was also measured	Demographic, anthropometric, diabetes duration, glycemic, lipid, comorbidities, lifestyle	7	Moderate
Tiantian et al (26), 2022	China	Wanfang	Cross-sectional	Hospital-based; Department of Geriatrics, Shengli Oilfield Central Hospital	450	42 / 408	≥60 years	AWGS 2014	ASMI, handgrip strength, and 6-m gait speed	Demographic, anthropometric, glycemic, lipid, comorbidities, lifestyle	7	Moderate
Lingyan et al (27), 2022	China	Wanfang	Cross-sectional	Hospital-based; Health Management Center, West China Hospital, Sichuan University	236	52 / 184	≥60 years	AWGS 2019	DXA for ASMI; handgrip strength; 6-m gait speed; SARC-F	Demographic, anthropometric, diabetes duration, glycemic, lipid, comorbidities, lifestyle	8	High
Chenqiao et al (28), 2022	China	Wanfang	Cross-sectional	Hospital-based; inpatients from the Department of Endocrinology, Nanping First Hospital Affiliated to Fujian Medical University	160	96 / 64	≥60 years	AWGS 2019	DXA-based skeletal muscle mass/ASMI; handgrip strength; 6-m gait speed	Demographic, anthropometric, diabetes duration, glycemic	7	Moderate
Yuchen et al (29), 2021	China	Wanfang	Cross-sectional	Hospital-based; inpatients from the Department of Geriatrics, Peking University People’s Hospital	190	43 / 147	≥65 years	AWGS 2019	BIA for ASMI; handgrip strength; 6-m gait speed	Demographic, anthropometric, glycemic, lipid, hematological/nutritional, comorbidities	7	Moderate
Shiyue et al (30), 2024	China	PubMed	Cross-sectional	Hospital-based; Department of Endocrinology and Metabolism, The First People’s Hospital of Longquanyi District, Chengdu	263	111 / 152	≥60 years	AWGS 2019	BIA using InBody S10 for ASMI; handgrip strength; SPPB physical performance assessment	Demographic, lifestyle, medication-related	7	Moderate
Shun Matsuura et al (31), 2022	Japan	PubMed	Cross-sectional	Outpatient-based; Fujieda Municipal General Hospital	234	58 / 176	≥60 years	AWGS 2019	BIA using InBody 270 for SMI; handgrip strength	Demographic, anthropometric, diabetes duration, glycemic, nutritional, comorbidities, medication-related	7	Moderate
Shariff-Ghazali Sazlina et al (32), 2020	Malaysia	PubMed	Cross-sectional	Community-based / primary care-based; two public primary care clinics in Malaysia	506	144 / 362	≥60 years	AWGS 2014	BIA using OMRON body composition monitor for skeletal muscle index; JAMAR handgrip strength; 6-m gait speed	Lipid, comorbidities, lifestyle, medication-related	7	Moderate
Mengzhao et al (33), 2019	China	PubMed	Cross-sectional	Hospital-based; inpatients from the Department of Endocrinology and Metabolism, the First Hospital of Jilin University	132	38 / 94	≥65 years	AWGS 2014	DXA for ASMI; handgrip strength; 6-m gait speed; calf circumference	Demographic, anthropometric, lipid, lifestyle, medication-related	7	Moderate
Ikumi Yanagita et al (34), 2019	Japan	PubMed	Cross-sectional	Hospital-based; outpatients and inpatients from Muta Hospital	108	38 / 70	≥65 years	AWGS 2014	BIA for skeletal muscle mass index; handgrip strength; walking speed	Demographic, anthropometric, diabetes duration, glycemic, hematological/nutritional, medication-related	6	Moderate
A.H.E. Küçükdiler et al (35), 2019	Türkiye	PubMed	Cross-sectional	Hospital-based; outpatient clinic of the Geriatric Medicine Department, Ankara University School of Medicine	60	30 / 30	≥65 years	EWGSOP1	BIA for skeletal muscle mass; handgrip strength; 4-m gait speed	Demographic, anthropometric, hematological, comorbidities	6	Moderate
Aslihan Calim et al (36), 2025	Türkiye	Embase	Cross-sectional	Hospital-based; Internal Medicine Department, Şişli Hamidiye Etfal Training and Research Hospital	292	65 / 227	≥60 years	EWGSOP2	Handgrip strength; calf circumference as muscle mass indicator; 4-m gait speed	Demographic, hematological, comorbidities, lifestyle, medication-related	7	Moderate
de Freitas et al (37), 2020	Brazil	PubMed	Cross-sectional	Outpatient-based; diabetes ambulatory care center of a public hospital in Porto Alegre	242	41 / 201	≥60 years	EWGSOP1	BIA using InBody 230 for muscle mass/SMI; Jamar handgrip strength; timed-up-and-go test; calf circumference also assessed	Demographic, anthropometric, glycemic, lipid, nutritional/vitamin D, comorbidities, lifestyle, medication-related	7	Moderate
Murata et al (38), 2018	Japan	PubMed	Cross-sectional	Outpatient-based; Clinical Center for Diabetes, Fuchu Hospital	288	44 / 244	≥65 years	AWGS 2014	BIA using Tanita MC780A for SMI; handgrip strength; 6-m walking speed	Demographic, anthropometric, diabetes duration, glycemic, nutritional, comorbidities	7	Moderate

### Methodological quality and risk of bias

The methodological quality of the 19 included cross-sectional studies was assessed using the AHRQ checklist. The total AHRQ scores ranged from 6 to 8, indicating overall moderate to high methodological quality. According to the predefined scoring criteria, three studies were rated as high quality and sixteen studies as moderate quality, with no low-quality studies identified. The total quality scores are summarized in **Table 1**, and the item-level AHRQ assessment is presented in **Supplementary Table 1**.

### Sensitivity analysis

Leave-one-out sensitivity analyses were performed for all outcomes included in the quantitative meta-analysis. The pooled estimates were generally stable for most outcomes, including age, BMI, diabetes duration, fasting blood glucose, HbA1c, total cholesterol, triglycerides, HDL-C, LDL-C, hemoglobin, serum albumin, hypertension, smoking exposure, insulin therapy, and coronary/ischemic heart disease. For smoking exposure, exclusion of Tiantian 2022 reduced heterogeneity to 0% without changing statistical significance, whereas the pooled result for 25(OH)D was sensitive to the exclusion of de Freitas 2020. The male-sex analysis also showed some sensitivity to the exclusion of an individual study. Detailed ranges of pooled estimates and I^2^ values from the leave-one-out analyses are presented in **Supplementary Table 2**. In addition, threshold-based sensitivity analyses were conducted by excluding studies with a minimum age criterion of ≥65 years. These analyses generally yielded findings consistent with the primary analyses, with the direction and statistical significance remaining unchanged for most outcomes. LDL-C was the only notable exception, becoming statistically significant after exclusion of the ≥65-year studies (MD = 0.10 mmol/L, 95% CI 0.04 to 0.17, P = 0.002; I^2^ = 37%); however, this result should be interpreted cautiously because only three studies remained in the analysis. Detailed results of the threshold-based sensitivity analyses are presented in **Supplementary Table 3**.

### Associated factors

#### Demographic characteristics

**Figure 2:**
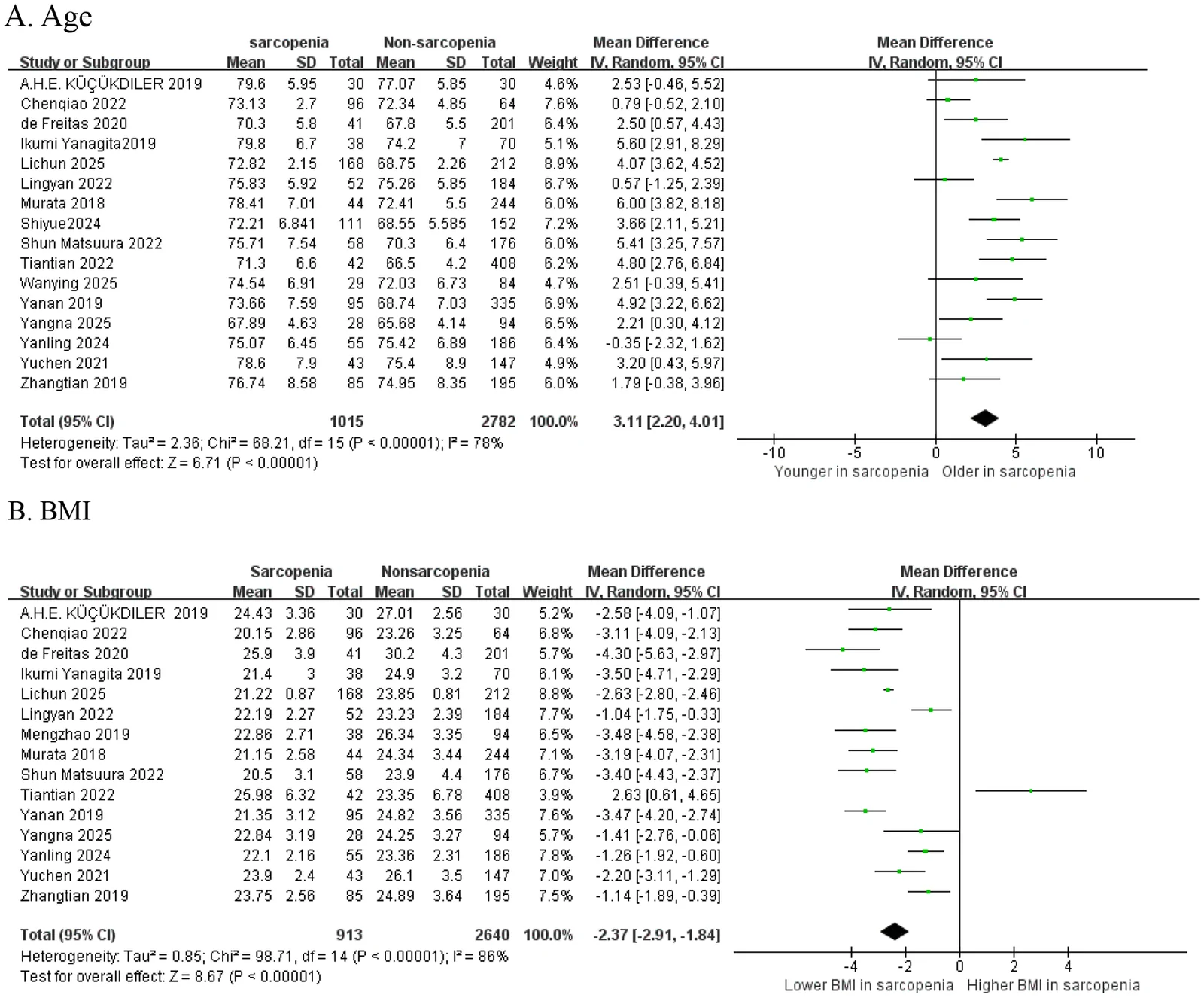
Forest plots of demographic and anthropometric factors associated with sarcopenia in older patients with type 2 diabetes mellitus. **(A)** Age. **(B)** Body mass index (BMI). MD, mean difference; CI, confidence interval.

**Figure 3:**
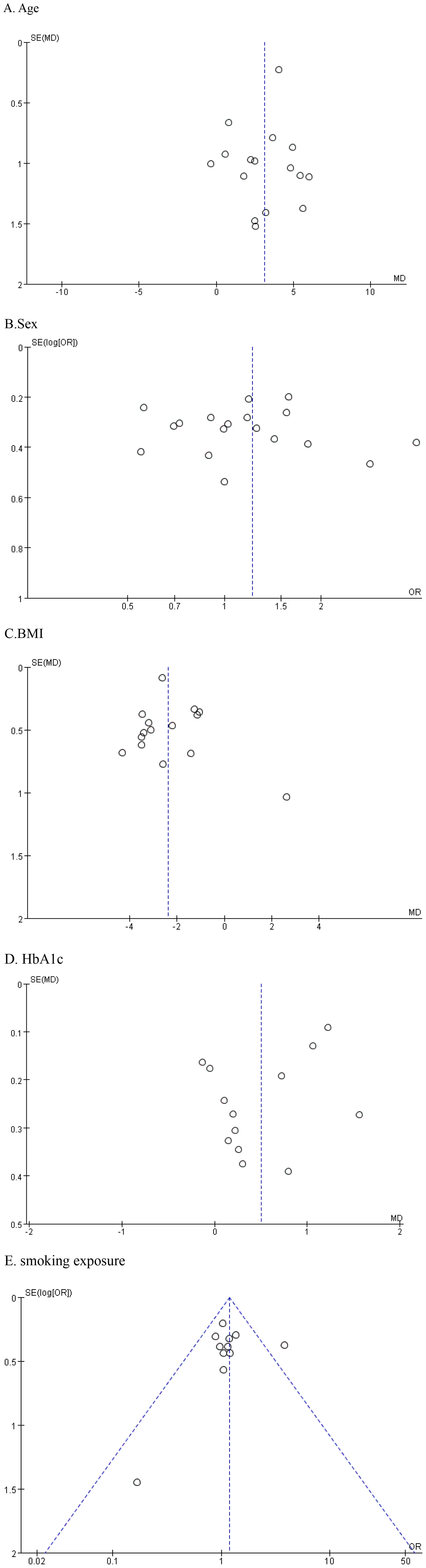
Funnel plots for the assessment of potential publication bias and small-study effects. **(A)** Age (Egger’s test, P = 0.259); **(B)** male sex (P = 0.434); **(C)** body mass index (BMI; P = 0.544); **(D)** glycated hemoglobin (HbA1c; P = 0.072); and **(E)** smoking exposure (P = 0.770). No statistically significant small-study effects were detected. SE, standard error; MD, mean difference; OR, odds ratio.

Demographic characteristics, including age and sex, were analyzed as factors associated with sarcopenia in older patients with type 2 diabetes mellitus. Sixteen studies (20–31, 34, 35, 37, 38) reported age data suitable for meta-analysis. The pooled result showed that patients with sarcopenia were significantly older than those without sarcopenia (as shown in **Figure 2A**) (MD = 3.11 years, 95% CI 2.20 to 4.01, P < 0.00001, τ^2^ = 2.36), with substantial heterogeneity across studies (I^2^ = 78%). In subgroup analysis restricted to studies using the AWGS 2014 or AWGS 2019 diagnostic criteria, the association between older age and sarcopenia remained significant in both the AWGS 2014 subgroup (MD = 4.17 years, 95% CI 2.77 to 5.57) and the AWGS 2019 subgroup (MD = 2.48 years, 95% CI 1.03 to 3.92). No statistically significant subgroup difference was observed between the two criteria-based subgroups (P for subgroup difference = 0.10). Visual inspection of the funnel plot showed an approximately symmetric distribution(as shown in **Figure 3A**), and Egger’s test did not indicate significant small-study effects (intercept = -1.06, P = 0.259).

Nineteen studies (20–38) reported sex distribution, with male sex treated as the event in the dichotomous analysis. The overall pooled result showed no statistically significant association between male sex and sarcopenia, although a positive trend was observed (OR = 1.22, 95% CI 0.96 to 1.55, P = 0.10; I^2^= 64%, τ^2^ = 0.17), with moderate heterogeneity. In subgroup analysis restricted to studies using AWGS criteria, no significant association was observed in either the AWGS 2014 subgroup (OR = 1.29, 95% CI 0.81 to 2.07) or the AWGS 2019 subgroup (OR = 1.09, 95% CI 0.89 to 1.34). There was no significant difference between the two subgroups (P for subgroup difference = 0.52). Funnel plot inspection showed no obvious asymmetry(as shown in **Figure 3B**), and Egger’s test also suggested no significant small-study effects (intercept = 1.19, P = 0.434). Overall, older age, rather than sex, appeared to be more consistently associated with sarcopenia in older adults with type 2 diabetes mellitus.

### Anthropometric indicators

Body mass index (BMI) was analyzed as the main anthropometric indicator associated with sarcopenia in older patients with type 2 diabetes mellitus. Fifteen studies reported BMI (20, 21, 23–29, 31, 33–35, 37, 38). Data suitable for meta-analysis. The pooled result showed that patients with sarcopenia had significantly lower BMI than those without sarcopenia(as shown in **Figure 2B**) (MD = -2.37 kg/m^2^, 95% CI -2.91 to -1.84, P < 0.00001, τ^2^ = 0.85), with substantial heterogeneity across studies (I^2^ = 86%). Subgroup analysis according to the diagnostic criteria for sarcopenia showed that the association between lower BMI and sarcopenia remained consistent in both the AWGS 2014 and AWGS 2019 subgroups, and no significant difference was observed between subgroups (P for subgroup difference = 0.67). Visual inspection of the funnel plot showed no obvious asymmetry(as shown in **Figure 3C**). Egger’s test also did not indicate significant small-study effects, with an intercept of 0.64 (standard error = 1.03, 95% CI -1.58 to 2.86, P = 0.544). Overall, lower BMI was significantly associated with sarcopenia in older adults with type 2 diabetes mellitus, although the substantial heterogeneity suggests that this finding should be interpreted with caution.

### Diabetes-related factors

**Figure 4:**
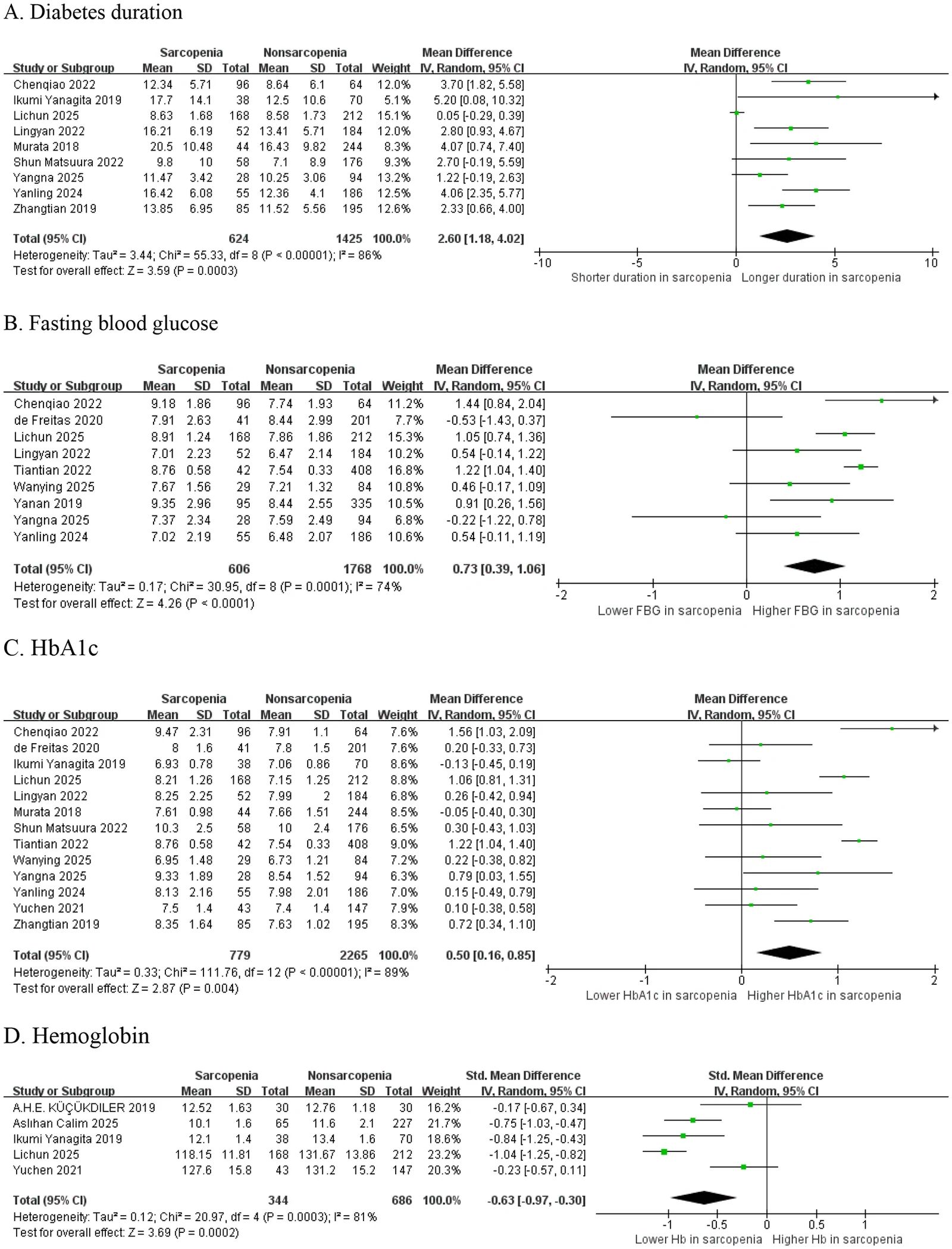
Forest plots of metabolic and hematological factors associated with sarcopenia in older patients with type 2 diabetes mellitus. **(A)** Diabetes duration. **(B)** Fasting blood glucose. **(C)** Glycated hemoglobin (HbA1c). **(D)** Hemoglobin (Hb).

Diabetes-related factors, including diabetes duration, fasting blood glucose (FBG), HbA1c, and insulin therapy, were analyzed to evaluate their associations with sarcopenia in older patients with type 2 diabetes mellitus. Nine studies (20, 21, 23, 25, 27, 28, 31, 34, 38) reported diabetes duration data suitable for meta-analysis. The pooled result showed that patients with sarcopenia had a significantly longer duration of diabetes than those without sarcopenia (as shown in **Figure 4A**) (MD = 2.60 years, 95% CI 1.18 to 4.02, P = 0.0003,I^2^ = 86%, τ^2^ = 3.44), with substantial heterogeneity across studies (I^2^ = 86%). Similarly, nine studies 20-22,24-28,37 reported FBG data, and the pooled result showed significantly higher FBG levels in the sarcopenia group than in the nonsarcopenia group (as shown in **Figure 4B**) (MD = 0.73 mmol/L, 95% CI 0.39 to 1.06, P < 0.0001, I^2^ = 74%, τ^2^ = 0.17), with high heterogeneity (I^2^ = 74%).

Thirteen studies (20–23, 25–29, 31, 34, 37, 38) reported HbA1c data suitable for meta-analysis. The pooled result showed that patients with sarcopenia had significantly higher HbA1c levels than those without sarcopenia (as shown in **Figure 4C**) (MD = 0.50%, 95% CI 0.16 to 0.85, P = 0.004; I^2^ = 89%, τ^2^ = 0.33), with substantial heterogeneity (I^2^ = 89%). Because more than ten studies were included in the HbA1c analysis, potential small-study effects were assessed using funnel plot inspection and Egger’s test. Visual inspection of the funnel plot suggested some degree of asymmetry (as shown in **Figure 3D**); however, Egger’s test did not reach statistical significance, with an intercept of -3.41 (standard error = 1.72, 95% CI -7.19 to 0.36, P = 0.072), indicating no statistically significant small-study effects. Nevertheless, given the borderline P value and substantial heterogeneity, this result should be interpreted cautiously.

Nine studies (24, 30–34, 36–38,) reported insulin therapy data. The pooled result showed no statistically significant association between insulin therapy and sarcopenia (OR = 1.09, 95% CI 0.89 to 1.33, P = 0.40), with low heterogeneity across studies (I^2^ = 26%). Overall, longer diabetes duration and poorer glycemic control, reflected by higher FBG and HbA1c levels, were significantly associated with sarcopenia in older adults with type 2 diabetes mellitus, whereas insulin therapy itself was not significantly associated with sarcopenia.

### Lipid metabolism indicators

Lipid metabolism indicators, including total cholesterol (TC), triglycerides (TG), high-density lipoprotein cholesterol (HDL-C), and low-density lipoprotein cholesterol (LDL-C), were analyzed to evaluate their associations with sarcopenia in older patients with type 2 diabetes mellitus. Six studies (20, 25–27, 29, 37) reported TC data suitable for meta-analysis. The pooled result showed no significant difference in TC levels between patients with and without sarcopenia (MD = -0.10 mmol/L, 95% CI -0.22 to 0.02, P = 0.11), with low-to-moderate heterogeneity across studies (I^2^ = 38%). Four studies (25–27, 32) reported TG data, and the pooled result also showed no significant difference between the sarcopenia and nonsarcopenia groups (MD = 0.02 mmol/L, 95% CI -0.04 to 0.08, P = 0.45), with no observed heterogeneity (I^2^ = 0%).

Seven studies (25–27, 33, 34, 37, 38) reported HDL-C data suitable for meta-analysis. HDL-C was assessed using the standardized mean difference because of differences in reporting units or measurement scales across studies. The pooled result showed no significant association between HDL-C and sarcopenia (SMD = 0.00, 95% CI −0.13 to 0.12, P = 0.99), with no observed heterogeneity (I^2^ = 0%). Five studies (25–27, 33, 38) reported LDL-C data suitable for meta-analysis. The pooled result showed no significant difference in LDL-C levels between patients with and without sarcopenia (MD = 0.05 mmol/L, 95% CI −0.06 to 0.15, P = 0.38; I^2^ = 55%, τ^2^ = 0.01), with moderate heterogeneity. Because fewer than ten studies were included for each lipid metabolism indicator, funnel plots and Egger’s tests were not performed. Overall, the available evidence did not show significant associations between lipid metabolism indicators and sarcopenia in older adults with type 2 diabetes mellitus.

### Nutritional and biochemical indicators

Nutritional and biochemical indicators, including hemoglobin, serum albumin, and serum 25-hydroxyvitamin D [25(OH)D], were analyzed to evaluate their associations with sarcopenia in older patients with type 2 diabetes mellitus. Five studies (21, 29, 34–36) reported hemoglobin data suitable for meta-analysis. The pooled result showed that patients with sarcopenia had significantly lower hemoglobin levels than those without sarcopenia (as shown in **Figure 4D**) (SMD = −0.63, 95% CI −0.97 to −0.30, P = 0.0002; I^2^ = 81%, τ^2^ = 0.12), with substantial heterogeneity across studies (I^2^ = 81%). This finding suggests that lower hemoglobin levels may be associated with sarcopenia in this population.

Six studies (21, 29, 31, 34, 37, 38) reported serum albumin data. The pooled result showed no statistically significant difference in serum albumin levels between the sarcopenia and nonsarcopenia groups (SMD = −0.28, 95% CI −0.75 to 0.19, P = 0.24; I^2^ = 93%, τ^2^ = 0.32), with substantial heterogeneity. Three studies 20,23,37 reported serum 25(OH)D data suitable for quantitative synthesis. Although patients with sarcopenia showed a trend toward lower 25(OH)D levels, the pooled result was not statistically significant (MD = −1.80, 95% CI −5.23 to 1.64, P = 0.31; I^2^ = 83%, τ^2^ = 7.54), with high heterogeneity (I^2^ = 83%). Sensitivity analysis suggested that the pooled result for 25(OH)D was influenced by de Freitas 2020; therefore, this finding should be interpreted cautiously.

Because fewer than ten studies were included for each nutritional and biochemical indicator, funnel plots and Egger’s tests were not performed. Overall, lower hemoglobin levels were significantly associated with sarcopenia, whereas serum albumin and 25(OH)D showed no statistically significant associations in the pooled analyses.

### Comorbidities and lifestyle-related factors

Comorbidities and lifestyle-related factors, including hypertension, smoking exposure, and coronary/ischemic heart disease, were analyzed to evaluate their associations with sarcopenia in older patients with type 2 diabetes mellitus. Eight studies (25–27, 29, 31, 32, 35, 36) reported hypertension data suitable for meta-analysis. The pooled result showed no statistically significant association between hypertension and sarcopenia (OR = 0.89, 95% CI 0.69 to 1.16, P = 0.40), with no observed heterogeneity across studies (I^2^ = 0%).

Eleven studies (20–22, 25–27, 30, 32, 33, 36, 37) reported smoking exposure data. The pooled result showed no statistically significant association between smoking exposure and sarcopenia, although a positive trend was observed (OR = 1.19, 95% CI 0.97 to 1.46, P = 0.09), with low heterogeneity (I^2^ = 26%). Because more than ten studies were included in the smoking exposure analysis, potential small-study effects were assessed using funnel plot inspection and Egger’s test. Visual inspection of the funnel plot showed no obvious asymmetry (as shown in **Figure 3E**), and Egger’s test did not indicate significant small-study effects, with an intercept of -0.34 (standard error = 1.13, 95% CI -2.89 to 2.21, P = 0.770).

Six studies (25, 27, 29, 32, 33, 36) reported coronary/ischemic heart disease data suitable for meta-analysis. The pooled result showed no statistically significant association between coronary/ischemic heart disease and sarcopenia, although a nonsignificant trend toward a higher prevalence was observed in patients with sarcopenia (OR = 1.27, 95% CI 0.96 to 1.69, P = 0.10), with no observed heterogeneity (I^2^ = 0%). Because fewer than 10 studies were included, funnel plot inspection and Egger’s test were not performed for this outcome.

### Narrative synthesis of additional factors

Additional factors that could not be quantitatively pooled included inflammatory markers, renal function, serum uric acid, insulin resistance-related indicators, diabetic microvascular complications, physical activity, and metformin/biguanide use. Quantitative pooling was not performed because of limited study numbers and heterogeneity in definitions, measurement methods, units, and reporting formats. Individual study results, including effect estimates, 95% CIs, P values, and adjusted or unadjusted estimates where available, are presented in **Supplementary Table 4**. Among these factors, lower physical activity showed the most consistent association with sarcopenia, whereas findings for the other factors were generally heterogeneous or inconsistent.

### Exploration of heterogeneity

Substantial or clinically important heterogeneity was observed for age, BMI, diabetes duration, fasting blood glucose, HbA1c, hemoglobin, serum albumin, and 25(OH)D. Exploratory REML analyses produced pooled estimates that were generally similar in direction and magnitude to those obtained in the primary RevMan analyses. The REML-based 95% prediction intervals crossed the null value for all eight outcomes: age, −0.56 to 6.78 years; BMI, −5.33 to 0.64 kg/m^2^; diabetes duration, −1.07 to 6.04 years; fasting blood glucose, −0.53 to 1.94 mmol/L; HbA1c, −0.65 to 1.66 percentage points; hemoglobin, −2.02 to 0.85; serum albumin, −1.75 to 1.19; and 25(OH)D, −49.54 to 46.06 ng/mL. The particularly wide prediction interval for 25(OH)D should be interpreted with considerable caution because only three studies were available.

Exploratory univariable meta-regression was conducted for age, BMI, HbA1c, and diabetes duration. For age, sarcopenia diagnostic criteria did not significantly moderate the between-group difference (overall P = 0.250; R^2^ = 7.20%). Geographic region was also not statistically significant, although non-Chinese studies showed a nonsignificant trend toward a larger age difference than Chinese studies (β = 1.84 years, 95% CI −0.29 to 3.96, P = 0.085; R^2^ = 15.64%). For BMI, diagnostic criteria, geographic region, and study-level mean age were not statistically significant moderators. For HbA1c, geographic region significantly moderated the effect estimate, with a smaller between-group difference in non-Chinese studies than in Chinese studies (β = −0.68 percentage points, 95% CI −1.28 to −0.07, P = 0.032; R^2^ = 39.32%), whereas diagnostic criteria and study-level mean age were not statistically significant. For diabetes duration, none of the examined moderators reached statistical significance. Substantial residual heterogeneity remained after meta-regression. Meta-regression was not performed for fasting blood glucose, hemoglobin, serum albumin, or 25(OH)D because of the limited number of available studies. Detailed REML-based pooled estimates, 95% prediction intervals, and complete results of the exploratory univariable meta-regression analyses are presented in **Supplementary Table 5**.

### Certainty of evidence

The GRADE assessment indicated that the certainty of evidence was low for HDL-C, hypertension, and insulin therapy and very low for all other pooled outcomes. The evidence was initially rated as low because all included studies were cross-sectional observational studies. Further downgrading was primarily attributable to substantial inconsistency across studies, limited numbers of studies for some outcomes, and imprecision reflected by wide confidence intervals or intervals crossing the null value. Risk-of-bias concerns were also related to the absence of temporality and the predominant reliance on unadjusted group-level estimates. Therefore, confidence in the reported associations is limited, and the true magnitude of these associations may be substantially different from the pooled estimates. The detailed judgments and reasons for downgrading are presented in **Table 2**.

**Table 2:** Summary of findings and GRADE certainty of evidence for pooled outcomes.

Outcome	No. of studies	Effect estimate	Risk of bias	Inconsistency	Indirectness	Imprecision	Publication bias	Overall certainty	Main reason for rating
Age	16	MD = 3.11 years (95% CI 2.20 to 4.01)	Not serious	Serious	Not serious	Not serious	Not detected	Very low	Further downgraded for serious inconsistency (I^2^ = 78%); the 95% prediction interval crossed the null value.
Male sex	19	OR = 1.22 (95% CI 0.96 to 1.55)	Not serious	Serious	Not serious	Serious	Not detected	Very low	Further downgraded for inconsistency and imprecision because the confidence interval crossed the null value.
BMI	15	MD = −2.37 kg/m^2^ (95% CI −2.91 to −1.84)	Not serious	Serious	Not serious	Not serious	Not detected	Very low	Further downgraded for serious inconsistency (I^2^ = 86%); the 95% prediction interval crossed the null value.
Diabetes duration	9	MD = 2.60 years (95% CI 1.18 to 4.02)	Not serious	Serious	Not serious	Not serious	Not assessed	Very low	Further downgraded for serious inconsistency (I^2^ = 86%); the 95% prediction interval crossed the null value. Publication bias could not be assessed because fewer than 10 studies were available.
Fasting blood glucose	9	MD = 0.73 mmol/L (95% CI 0.39 to 1.06)	Not serious	Serious	Not serious	Not serious	Not assessed	Very low	Further downgraded for high inconsistency; the 95% prediction interval crossed the null value. Publication bias could not be assessed because fewer than 10 studies were available.
HbA1c	13	MD = 0.50 percentage points (95% CI 0.16 to 0.85)	Not serious	Serious	Not serious	Not serious	Possible	Very low	Further downgraded for serious inconsistency (I^2^ = 89%); the 95% prediction interval crossed the null value. Egger’s test was borderline (P = 0.072).
Total cholesterol	6	MD = −0.10 mmol/L (95% CI −0.22 to 0.02)	Not serious	Not serious	Not serious	Serious	Not assessed	Very low	Further downgraded for imprecision and limited evidence because only six studies were available and the confidence interval crossed the null value.
Triglycerides	4	MD = 0.02 mmol/L (95% CI −0.04 to 0.09)	Not serious	Not serious	Not serious	Serious	Not assessed	Very low	Further downgraded for imprecision and limited evidence because only four studies were available and the confidence interval crossed the null value.
HDL-C	9	SMD = 0.00 (95% CI −0.13 to 0.12)	Not serious	Not serious	Not serious	Not serious	Not assessed	Low	No further downgrading: the estimate was relatively precise and no heterogeneity was observed.
LDL-C	5	MD = 0.05 mmol/L (95% CI −0.06 to 0.15)	Not serious	Serious	Not serious	Serious	Not assessed	Very low	Further downgraded for inconsistency and imprecision because only five studies were available and the confidence interval crossed the null value.
Hemoglobin	5	SMD = −0.63 (95% CI −0.97 to −0.30)	Not serious	Serious	Not serious	Serious	Not assessed	Very low	Further downgraded for serious inconsistency (I^2^ = 81%) and limited evidence; only five studies were available and the 95% prediction interval crossed the null value.
Serum albumin	6	SMD = −0.28 (95% CI −0.75 to 0.19)	Not serious	Serious	Not serious	Serious	Not assessed	Very low	Further downgraded for serious inconsistency (I^2^ = 93%) and imprecision because the confidence interval and prediction interval crossed the null value.
25(OH)D	3	MD = −1.80 ng/mL (95% CI −5.23 to 1.64)	Not serious	Serious	Not serious	Serious	Not assessed	Very low	Further downgraded for serious inconsistency and imprecision: only three studies were available, the prediction interval was extremely wide, and the result was sensitive to exclusion of de Freitas 2020.
Hypertension	8	OR = 0.89 (95% CI 0.69 to 1.16)	Not serious	Not serious	Not serious	Not serious	Not assessed	Low	No further downgrading: no heterogeneity was observed and no serious additional limitation was identified.
Smoking exposure	11	OR = 1.19 (95% CI 0.97 to 1.46)	Not serious	Not serious	Not serious	Serious	Not detected	Very low	Further downgraded for imprecision because the confidence interval crossed the null value; sensitivity analysis reduced heterogeneity without changing the effect direction.
Insulin therapy	9	OR = 1.09 (95% CI 0.89 to 1.33)	Not serious	Not serious	Not serious	Not serious	Not assessed	Low	No further downgrading: heterogeneity was low (I^2^ = 26%) and no serious additional limitation was identified.
Coronary/ischemic heart disease	6	OR = 1.27 (95% CI 0.96 to 1.69)	Not serious	Not serious	Not serious	Serious	Not assessed	Very low	Further downgraded for imprecision and limited evidence because only six studies were available and the confidence interval crossed the null value.

## Discussion

This systematic review and meta-analysis included 19 cross-sectional studies involving 4,727 older patients with type 2 diabetes mellitus. The findings showed that older age, lower BMI, longer diabetes duration, higher fasting blood glucose, higher HbA1c, and lower hemoglobin levels were significantly associated with sarcopenia. In contrast, male sex, total cholesterol, triglycerides, HDL-C, LDL-C, serum albumin, 25(OH)D, hypertension, smoking exposure, insulin therapy, and coronary/ischemic heart disease were not significantly associated with sarcopenia in the pooled analyses. These results suggest that sarcopenia in older adults with type 2 diabetes mellitus is more closely related to aging, poor glycemic control, reduced body reserve, and impaired hematological or nutritional status than to lipid metabolism indicators or common comorbidities alone. The present findings are broadly consistent with previous meta-analyses regarding the associations of older age, poorer glycemic control, and lower BMI with sarcopenia. However, earlier reviews often included broader age ranges or focused mainly on Asian populations. In contrast, our review focused exclusively on adults aged ≥60 years, included studies using both AWGS and EWGSOP criteria, and quantitatively synthesized a wider range of clinical indicators. To our knowledge, this study provides the first pooled estimate of the association between hemoglobin and sarcopenia specifically in older patients with T2DM.

Age was the most consistent demographic factor associated with sarcopenia. The pooled analysis showed that patients with sarcopenia were significantly older than those without sarcopenia. This finding is biologically plausible, as aging is accompanied by progressive declines in muscle mass, muscle strength, mitochondrial function, neuromuscular integrity, and muscle regenerative capacity (39). In older patients with type 2 diabetes mellitus, these age-related changes may be further aggravated by chronic hyperglycemia, insulin resistance, oxidative stress, and low-grade inflammation. Subgroup analyses based on AWGS 2014 and AWGS 2019 criteria showed that the association between older age and sarcopenia remained significant in both subgroups, suggesting that the finding was relatively robust across diagnostic criteria. In contrast, male sex was not significantly associated with sarcopenia, indicating that sex alone was not consistently associated with sarcopenia in this population.

lower BMI may reflect reduced body reserve and was significantly associated with sarcopenia in older adults with type 2 diabetes mellitus. Although BMI cannot distinguish muscle mass from fat mass, a lower BMI in this population may reflect insufficient nutritional reserve, weight loss, reduced muscle mass, or frailty-related body composition changes (40, 41) This result also highlights the need to avoid focusing only on obesity and metabolic excess in older diabetic patients, as undernutrition and muscle depletion may coexist with diabetes and be associated with poorer physical function. However, the heterogeneity of BMI was substantial, which may be related to differences in population characteristics, ethnicity, baseline body composition, diagnostic criteria for sarcopenia, and study setting.

Diabetes-related factors showed clear associations with sarcopenia. Patients with sarcopenia had longer diabetes duration, higher fasting blood glucose, and higher HbA1c levels than those without sarcopenia. These findings indicate that longer diabetes duration and poorer glycemic control were associated with sarcopenia. Possible biological explanations proposed in previous mechanistic studies include the accumulation of advanced glycation end products, oxidative stress, mitochondrial dysfunction, and impaired anabolic signaling. Chronic hyperglycemia can promote the accumulation of advanced glycation end products, oxidative stress, mitochondrial dysfunction, endothelial dysfunction, and inflammatory activation, all of which may impair muscle protein synthesis and accelerate muscle protein degradation (42, 43). Insulin resistance may further suppress the PI3K-AKT-mTOR anabolic pathway and enhance catabolic pathways such as the ubiquitin-proteasome system, thereby promoting loss of muscle mass and function (44). Prolonged diabetes duration may serve as a marker of cumulative metabolic burden, although the present findings do not establish that longer disease duration causally leads to sarcopenia. These complex metabolic interactions highlight the value of integrative and genetically informed analytical approaches to disentangle the biological pathways linking chronic hyperglycemia and muscle decline (45–47). These observations are consistent with the concept that diabetes and sarcopenia may interact in a vicious cycle (48). In contrast, insulin therapy itself was not significantly associated with sarcopenia. This result should be interpreted cautiously, because insulin use may reflect diabetes severity, longer disease duration, poorer β-cell function, or more complex treatment regimens rather than a direct biological effect of insulin on muscle.

Among lipid metabolism indicators, TC, TG, HDL-C, and LDL-C were not significantly different between patients with and without sarcopenia. Previous studies have proposed that dyslipidemia may contribute to muscle dysfunction through lipotoxicity, ectopic lipid deposition, endothelial dysfunction, oxidative stress, and chronic inflammation (49–51). In particular, LDL-C and oxidized LDL have been implicated in impaired microvascular perfusion, reduced nitric oxide bioavailability, and inflammatory activation, which may theoretically affect nutrient and oxygen delivery to skeletal muscle (52). However, the present pooled results did not support a significant association between lipid parameters and sarcopenia in older patients with type 2 diabetes mellitus. This discrepancy may be partly explained by differences in lipid-lowering medication use, dietary patterns, obesity status, glycemic control, and comorbidity burden across studies. Therefore, the current pooled evidence did not support consistent associations between conventional lipid indicators and sarcopenia in this population.

Regarding nutritional and biochemical indicators, lower hemoglobin was significantly associated with sarcopenia. This finding suggests that anemia or reduced oxygen-carrying capacity may be related to muscle deterioration in older diabetic patients. Low hemoglobin may reduce oxygen delivery to skeletal muscle, impair mitochondrial oxidative metabolism, promote fatigue, and contribute to reduced physical activity, thereby accelerating the decline in muscle mass and function (53, 54). Previous evidence has also indicated an association between low hemoglobin levels and sarcopenia, although older patients with type 2 diabetes mellitus have not always been analyzed as a specific subgroup (55). The present study therefore extends existing evidence by suggesting that hemoglobin may be a clinically useful and easily accessible marker associated with sarcopenia in this population. In contrast, serum albumin was not significantly associated with sarcopenia, despite a nonsignificant trend toward lower levels in the sarcopenia group. The high heterogeneity observed for serum albumin may be related to differences in nutritional status, inflammation, renal function, liver function, and acute or chronic disease burden. Similarly, 25(OH)D showed a nonsignificant trend toward lower levels in patients with sarcopenia, but the pooled result was not statistically significant and was sensitive to the exclusion of de Freitas 2020. Therefore, the association between vitamin D status and sarcopenia in older patients with type 2 diabetes mellitus remains uncertain and requires further investigation.

Comorbidities and lifestyle-related factors showed limited associations with sarcopenia in the pooled analyses. Hypertension was not significantly associated with sarcopenia, and no heterogeneity was observed. Smoking exposure showed a positive but nonsignificant trend, and sensitivity analysis suggested that the result was partly influenced by Tiantian 2022, but the overall direction of association remained unchanged. Coronary/ischemic heart disease also showed a nonsignificant trend toward higher prevalence in the sarcopenia group. These findings suggest that although cardiovascular comorbidities and smoking may be clinically relevant in older diabetic patients, the current evidence is insufficient to confirm their significant association with sarcopenia. Moreover, these variables may be strongly confounded by age, sex, diabetes duration, metabolic control, physical activity, and overall health status.

The narrative synthesis of additional factors showed that lower physical activity was relatively consistently associated with sarcopenia, whereas findings for inflammatory markers, renal function, serum uric acid, insulin resistance-related indicators, diabetic microvascular complications, and metformin/biguanide use were generally heterogeneous or inconsistent and should therefore be interpreted cautiously.

The sensitivity analyses generally supported the robustness of the main findings. The associations of older age, lower BMI, longer diabetes duration, higher fasting blood glucose, higher HbA1c, and lower hemoglobin with sarcopenia remained directionally stable after sequential exclusion of individual studies. For several outcomes, such as diabetes duration and smoking exposure, exclusion of influential studies reduced heterogeneity without changing the overall interpretation. However, the result for 25(OH)D was sensitive to the exclusion of de Freitas 2020, indicating that this finding should be interpreted cautiously. Funnel plot inspection and Egger’s tests were performed only for outcomes with at least ten studies. No statistically significant small-study effects were detected for age, sex, BMI, HbA1c, or smoking exposure, although the HbA1c result showed a borderline Egger’s test P value and should be interpreted with caution.

The definition of older adulthood varies across countries, regions, and healthcare systems. Although ≥60 years was prespecified as the eligibility threshold in this review, six studies used the more restrictive threshold of ≥65 years. Threshold-based sensitivity analyses generally supported the robustness of the main findings, suggesting that differences in age definitions were unlikely to substantially affect most pooled associations. However, heterogeneity decreased for HbA1c and hemoglobin, and the LDL-C result became statistically significant after excluding studies using the ≥65-year threshold. These findings suggest that age-threshold differences may influence some outcomes, although the small number of remaining studies warrants cautious interpretation. Variation in sarcopenia diagnostic criteria and muscle assessment methods may also have contributed to between-study variability. AWGS 2014, AWGS 2019, EWGSOP1, and EWGSOP2 differ in their diagnostic algorithms and cutoff values and may therefore identify partially different patient populations. However, subgroup analyses and exploratory meta-regression did not identify diagnostic criteria as a significant moderator of the examined associations, although the small number of EWGSOP-based studies limits firm conclusions. In addition, muscle mass was assessed mainly by BIA, with some studies using DXA or calf circumference; differences between these methods may influence muscle mass estimates and consequently sarcopenia classification.

Substantial heterogeneity was observed for age, BMI, diabetes duration, fasting blood glucose, HbA1c, hemoglobin, serum albumin, and 25(OH)D. Exploratory meta-regression did not identify sarcopenia diagnostic criteria as a statistically significant moderator for age, BMI, HbA1c, or diabetes duration. Geographic region significantly moderated the HbA1c effect estimates, whereas only nonsignificant trends were observed for age and BMI. Study-level mean age was not a statistically significant moderator of BMI, HbA1c, or diabetes duration. These findings indicate that the prespecified study-level characteristics explained only a limited proportion of the observed heterogeneity. The age-threshold sensitivity analyses further suggested that differences in the operational definition of older adulthood were unlikely to be the predominant source of heterogeneity for most outcomes, although they may have contributed partly to the heterogeneity observed for HbA1c and hemoglobin. The remaining heterogeneity may reflect differences in study setting, participant selection, baseline body composition, nutritional status, glycemic management, diabetes complications, medication use, ethnicity, body-composition assessment methods, and laboratory measurement procedures. However, formal analyses of several of these variables were not feasible because they were inconsistently reported and some categories contained too few studies. All 95% prediction intervals crossed the null value. Therefore, although the average pooled associations for age, BMI, diabetes duration, fasting blood glucose, HbA1c, and hemoglobin were statistically significant, their magnitude and even their presence may vary across future comparable study settings. Substantial heterogeneity consequently reduces the consistency, generalizability, and certainty of the pooled estimates. Nevertheless, quantitative pooling was retained because the included studies addressed the same clinical question, compared sarcopenia and non-sarcopenia groups, and evaluated clinically comparable outcomes whose units could be harmonized or standardized. Random-effects models were therefore considered appropriate for estimating average associations across heterogeneous study settings. The pooled estimates should consequently be interpreted as average associations rather than effects that are necessarily present in every population or clinical setting. Translating such population-averaged associations into reliable individual-level predictions requires rigorous prognostic model development and external validation across diverse populations and healthcare settings (13–15). Prediction intervals based on very few studies, particularly that for 25(OH)D, should be interpreted with additional caution.

The GRADE assessment indicated low to very low certainty across all pooled outcomes. Although statistically significant average associations were observed for age, BMI, diabetes duration, fasting blood glucose, HbA1c, and hemoglobin, the low or very low certainty indicates limited confidence that the pooled estimates are close to the true associations. This uncertainty primarily reflects the cross-sectional nature of the evidence, the predominant use of unadjusted group-level estimates, substantial between-study heterogeneity, and imprecision for several outcomes. Consequently, the findings should be interpreted as hypothesis-generating associations rather than established independent predictors or causal determinants. Future prospective cohort studies with standardized sarcopenia definitions, consistent measurement methods, and adequate control of confounding may materially change both the magnitude of the estimated associations and confidence in the conclusions. Such prospective designs are essential for developing and validating machine learning-based risk prediction tools and predictive risk assessment frameworks for sarcopenia in chronic disease populations (16– 19).

Clinically, the identified factors should be regarded as cues for increased clinical vigilance rather than criteria for selective sarcopenia screening. Given the low to very low certainty of evidence and the cross-sectional nature of the included studies, poor glycemic control, lower BMI, or lower hemoglobin should not be used as stand-alone screening thresholds. Accordingly, Current guidelines recommend routine sarcopenia assessment in older adults with diabetes, with tools such as SARC-F or SARC-CalF for case finding, followed by assessment of muscle strength, physical performance, and muscle mass when indicated (56). The rationale for sarcopenia assessment is further supported by its prognostic relevance. Prospective studies in older adults with T2DM have linked sarcopenia with severe disability, rehospitalization, and all-cause mortality (57, 58), while broader evidence in older populations supports associations with falls and fractures. These findings support early recognition of sarcopenia, although prognostic evidence should be distinguished from the cross-sectional associations examined in the present meta-analysis.

Several limitations should be acknowledged. First, all included studies were cross-sectional, which precluded the establishment of temporal or causal relationships. Therefore, the identified factors should be interpreted as associated factors rather than independent risk factors or predictors. Methodological guidance on prognostic model development emphasizes that cross-sectional associations alone are insufficient for establishing validated predictive algorithms without temporal validation and external testing (14, 15). Second, most pooled estimates were derived from unadjusted group-level data. Although some studies reported multivariable-adjusted estimates, these were not consistently available and were based on substantially different covariate-adjustment models, preventing their quantitative synthesis. Consequently, residual confounding by factors such as age, sex, nutritional status, comorbidities, and medication use cannot be excluded. Third, substantial heterogeneity was observed for several outcomes, including age, BMI, diabetes duration, fasting blood glucose, HbA1c, hemoglobin, serum albumin, and 25(OH)D. Exploratory univariable meta-regression was feasible only for age, BMI, HbA1c, and diabetes duration. Sarcopenia diagnostic criteria were not statistically significant moderators of these outcomes, whereas geographic region significantly moderated only the HbA1c effect estimate. The age analysis showed nonsignificant geographic variation, and substantial residual heterogeneity remained after all meta-regression analyses. Because the analyses were based on aggregate study-level data, ecological bias cannot be excluded, and the observed moderator associations cannot be directly extrapolated to individual participants. Moreover, several moderator categories contained few studies, and multiple exploratory analyses were conducted; therefore, the statistically significant regional finding for HbA1c should be interpreted cautiously. Fourth, meta-regression was not performed for fasting blood glucose, hemoglobin, serum albumin, or 25(OH)D because the number of available studies was insufficient to support reliable moderator estimates.

Although prediction intervals were calculated, all crossed the null value, indicating considerable uncertainty regarding the effects expected in future comparable settings. Prediction intervals derived from very few studies, particularly the interval for 25(OH)D, were extremely imprecise. Substantial residual heterogeneity and the limited number of studies for several outcomes reduce the generalizability and certainty of the pooled findings.

Fifth, most studies were conducted in Asian populations, particularly in China, which may limit the generalizability of the findings to other ethnic and geographic populations. Finally, several potentially relevant factors, including inflammatory markers, renal function, serum uric acid, insulin resistance-related indicators, diabetic microvascular complications, physical activity, and metformin/biguanide use, were reported in only a limited number of studies or were assessed using heterogeneous definitions, measurement methods, units, and reporting formats, which precluded reliable quantitative pooling. These factors were therefore synthesized narratively using individual-study results, but the limited and heterogeneous evidence restricts the strength of conclusions that can be drawn. Other potentially relevant factors, such as dietary intake, protein consumption, and frailty, were also inconsistently reported across the included studies. Employing integrative analytical strategies that jointly model clinical, metabolic, and genetic data may help overcome these reporting gaps and clarify the multifactorial etiology of diabetic sarcopenia (46, 47). These limitations were reflected in the low to very low GRADE certainty ratings and substantially restrict the strength of the clinical inferences that can be drawn from the pooled estimates.

## Conclusion

In conclusion, older age, lower BMI, longer diabetes duration, higher fasting blood glucose, higher HbA1c, and lower hemoglobin levels were significantly associated with sarcopenia in older patients with type 2 diabetes mellitus. However, the certainty of evidence for all pooled outcomes was low to very low. Therefore, confidence in the magnitude and consistency of these associations remains limited, and future well-designed studies may materially change the current estimates. The identified factors may heighten clinical suspicion during routine, guideline-based sarcopenia assessment but should not be regarded as independent predictors, validated screening thresholds, or evidence of causal relationships. Prospective cohort studies using standardized sarcopenia diagnostic and measurement methods, with comprehensive adjustment for confounding, are required to confirm the temporal relationships and clinical utility of these associations.

## Data Availability

The original contributions presented in the study are included in the article/**Supplementary Material**. Further inquiries can be directed to the corresponding author.
